# Nodule‐type pneumonitis mimicking lung abscess or tumor recurrence on chest computed tomography identified as osimertinib‐induced pneumonitis: A case report

**DOI:** 10.1111/1759-7714.14682

**Published:** 2022-10-08

**Authors:** Ayumu Otsuki, Yuya Homma, Michinori Yoshimi, Hiroyuki Ito, Kei Nakashima

**Affiliations:** ^1^ Kameda Medical Center Kamogawa Japan

**Keywords:** abscess, lung cancer, pneumonitis

## Abstract

Osimertinib‐induced pneumonitis usually manifests as ground glass opacities (GGO) on chest computed tomography (CT) scans. Here, we encountered a case involving a 42‐year‐old woman who had nodule‐type pneumonitis mimicking lung abscess or tumor recurrence caused by osimertinib. When a nodular pattern is seen on the chest CT scan of a patient receiving osimertinib treatment, drug‐induced pneumonitis should be considered as a differential diagnosis. To the best of our knowledge, this is the first case of osimertinib‐induced pneumonitis that manifested as a nodule‐type pneumonitis mimicking lung abscess or tumor recurrence on chest CT scan.

## Graphical abstract

Osimertinib‐induced pneumonitis can manifest as a nodule‐type pneumonitis that mimics abscess or tumor recurrence. Early bronchoscopy must be performed to determine the precise duration of osimertinib treatment necessary for avoiding the development of pneumonitis and the change to next chemotherapy.

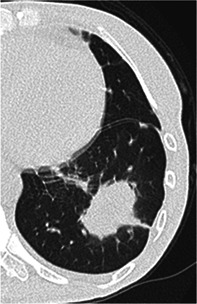

## INTRODUCTION

Epidermal growth factor receptor (EGFR) mutation is the most common mutation in non‐small cell lung cancer (NSCLC). Osimertinib is recommended as first‐line therapy for *EGFR*‐mutant NSCLC.^1^ Drug‐induced interstitial lung disease (DILD) is a side effect of EGFR‐tyrosine kinase inhibitors, including osimertinib. The incidence of osimertinib‐induced pneumonitis, a DILD, is 4%.^1^ Various manifestations of osimertinib‐induced pneumonitis have been reported.^2^, ^3^, ^4^, ^5^ However, to the best of our knowledge, nodule‐type pneumonitis mimicking lung abscess or tumor recurrence on chest computed tomography (CT) scan has not previously been reported. Here, we report a case of DILD caused by osimertinib in which a nodule pattern was observed on chest CT scan.

## CASE REPORT

**FIGURE 1:**
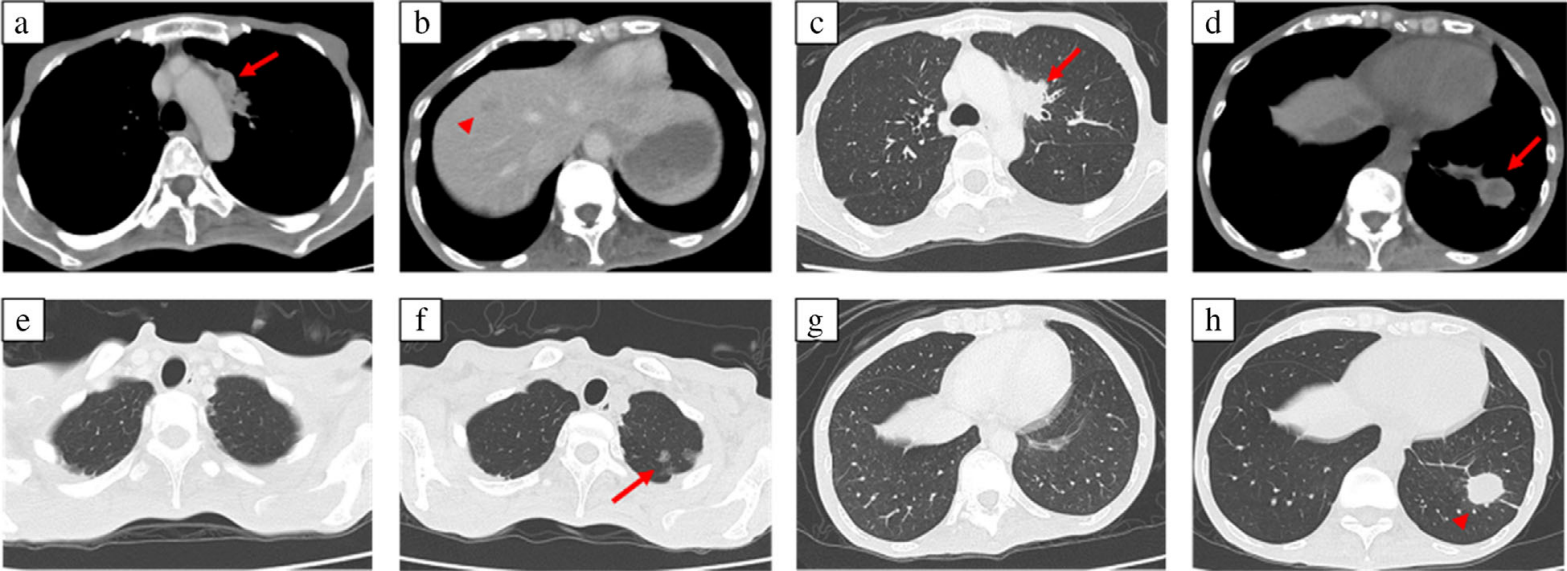
Whole‐body computed tomography (CT) scans. (a, b) The mediastinal window shows lymph node metastasis (arrow) and hepatic metastasis (arrowhead) before treatment. (c) The parenchymal window shows lung tumor before treatment (arrow). (d) The mediastinal window shows a low‐density area mimicking lung abscess or tumor recurrence on day 101. (arrow). (f, h) The parenchymal window shows ground glass opacity (arrow) and a nodule (arrowhead), which indicate pneumonitis caused by osimertinib on day 101, compared to (e, g) chest CT before treatment.

**FIGURE 2:**
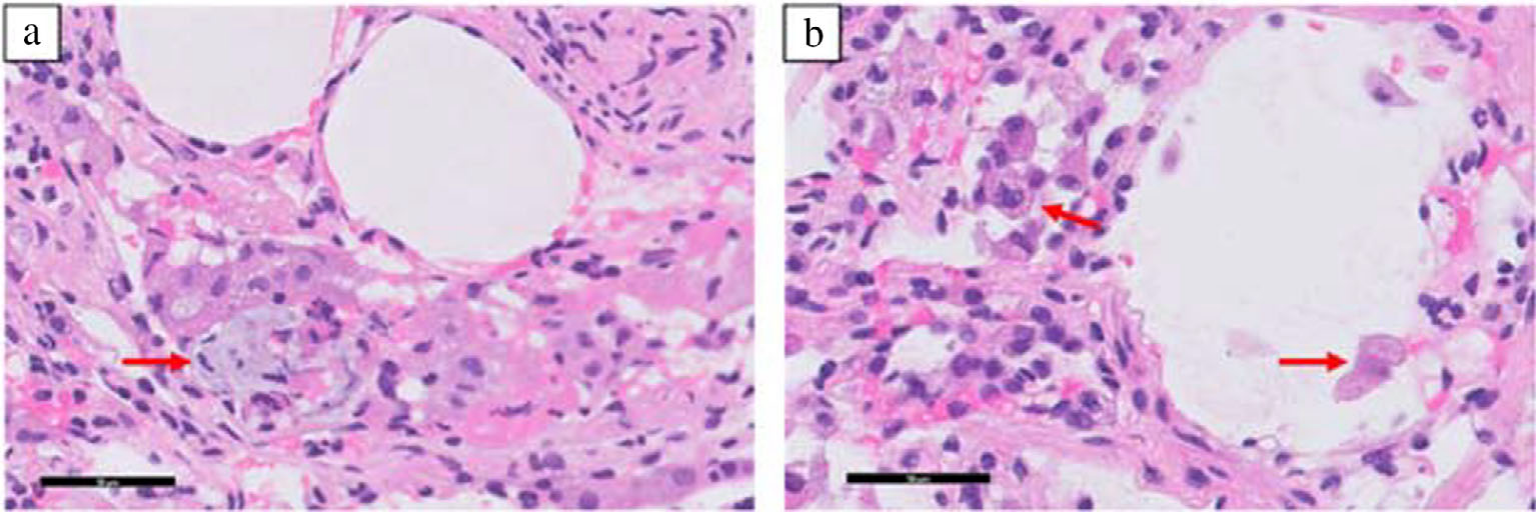
Findings of transbronchial lung biopsy showing organization with (a) fibrin (arrow) as well as (b) foamy macrophages and disruption of alveolar epithelial cells (arrow) (hematoxylin and eosin stain; ×400 magnification).

**FIGURE 3:**
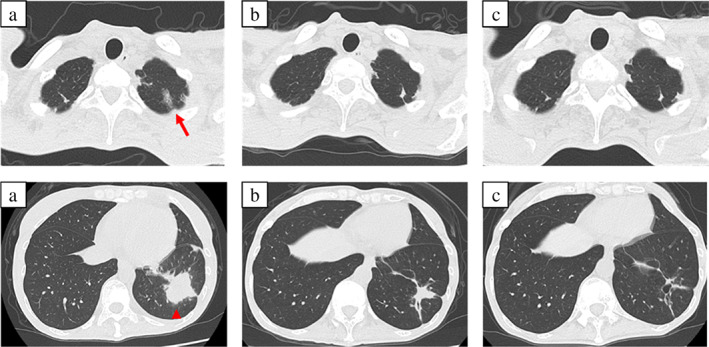
Chest computed tomography (CT) scans taken on (a) day 115, (b) day 165, and (c) day 193. Ground‐glass opacity (arrow) became organized and the nodule (arrowhead) disappeared during treatment with osimertinib.

A 42‐year‐old woman visited an orthopedist with complaints of back pain for 7 months and loss of appetite for 2 months. A computed tomography (CT) scan of her chest revealed a pulmonary nodule in the left upper lobe. Positron emission tomography (PET) findings led to a suspected diagnosis of lung cancer. She visited our institution for further examination. She had no history of smoking. Her Eastern Cooperative Oncology Group performance status (PS) score was 1 because of her back pain and emaciation (height: 155.5 cm, weight: 32.3 kg). Based on transbronchial lung biopsy findings, whole‐body CT scan, and head magnetic resonance imaging, she was diagnosed with c‐stage IVB lung adenocarcinoma (c‐T1cN2M1c OSS, HEP, BRA, OTH) (carcinoembryonic antigen [CEA], 55.9 ng/ml; sialylated carbohydrate antigen [KL‐6], 5833.4 U/ml; Figure 1). She received palliative radiotherapy for the lumbar spine (30 Gy/10 Fr) from day 3 and whole‐brain irradiation (30 Gy/10 Fr) from day 7 until her histology and driver mutation analysis results were obtained, which indicated an *EGFR* mutation (exon 19 deletion). She started taking osimertinib 80 mg as first‐line therapy from day 33. When she developed grade 3 leukopenia (Common Terminology Criteria for Adverse Events, version 5.0), osimertinib treatment was terminated. The leukopenia improved by day 52, and osimertinib 40 mg was resumed on day 53. Her PS improved by day 59 (CEA, 47.8 ng/ml; carbohydrate antigen 19‐9 [CA19‐9], 70.0 U/ml; KL‐6, 2664.6 U/ml). On day 101 (CEA, 17.8 ng/ml; CA19‐9, 24.5 U/ml; KL‐6, 1174.9 U/ml), a chest CT scan revealed ground‐glass opacities (GGO) in the upper lobe of the left lung and a nodule in the lower lobe of the left lung, and the mediastinal window showed low‐density areas, suggestive of abscesses (Figure 1). She had no symptoms and continued taking osimertinib. We suspected tumor resistance to osimertinib rather than an abscess because she had no fever, and her white blood cell count and C‐reactive protein levels were 6700/μl and 0.77 mg/dl, respectively. The osimertinib dose was then increased to 80 mg. We performed transbronchial biopsy of the nodule on day 107. Pathological evaluation of the specimens revealed acute lung injury (Figure 2), and tissue cultures returned negative results. She was diagnosed with osimertinib‐induced pneumonitis and stopped taking osimertinib, following which the nodule enlarged on day 115. Chest CT scans showed gradual recovery of GGO and the nodule, but on day 165, the tumor grew and other laboratory findings worsened (CEA, 15.0 ng/ml; CA19‐9, 73.3 U/ml; KL‐6, 1328.6 U/ml). Consequently, osimertinib 40 mg treatment, along with prednisolone 10 mg, was resumed; subsequently, tumor growth decreased over a month (CEA, 6.9 ng/ml; CA19‐9, 25.5 U/ml; KL‐6, 843.9 U/ml on day 193), and the nodule almost disappeared (Figure 3).

## DISCUSSION

Osimertinib‐induced pneumonitis, an adverse event, is rarely serious or fatal. Previous studies have reported GGO‐type pneumonitis, and a case study has discussed small nodule‐type pneumonitis.^2^, ^3^, ^4^, ^5^ However, there are no reports of nodule‐type pneumonitis mimicking lung abscess or tumor recurrence on chest CT scan, identified as osimertinib‐induced lung pneumonitis. Interestingly, osimertinib rechallenge by administering both osimertinib and low‐dose prednisolone was successful. Most prior studies have reported a successful osimertinib rechallenge with an initial prednisolone dose of 0.5 mg/kg.^6^, ^7^ The etiology of the new nodule on chest CT scan remains unknown as biopsy was not performed on the nodule. For proper management, clinicians should be aware that nodule‐type pneumonitis can occur during osimertinib treatment in patients with lung cancer. Early bronchoscopic biopsy is crucial for diagnosing interstitial pneumonitis due to osimertinib. In some cases, patients continue taking osimertinib, and it prolongs their survival.

In conclusion, osimertinib‐induced pneumonitis should be considered in the differential diagnosis when a new nodule develops after osimertinib administration in patients with lung cancer. Diagnosis with transbronchial biopsy is important to determine the etiology of the nodule for appropriate management in these patients.

## AUTHOR CONTRIBUTIONS

AO designed and supervised this manuscript and collected data. YH collected and managed data. MY made a provision of materials of this manuscipt. HI and KN gave helpful suggestions and reviewed the manuscript. All authors read and approved the final manuscript.

## CONFLICT OF INTEREST

The authors declare no conflict of interest.
